# Differential Effects of BMI on Brain Response to Odor in Olfactory, Reward and Memory Regions: Evidence from fMRI

**DOI:** 10.3390/nu11040926

**Published:** 2019-04-24

**Authors:** Aaron Jacobson, Erin Green, Lori Haase, Jacquelyn Szajer, Claire Murphy

**Affiliations:** 1 Department of Psychology, San Diego State University, San Diego, CA 92182, USA; adjacobson1@gmail.com; 2 San Diego State University/University of California San Diego Joint Doctoral Program in Clinical Psychology, San Diego, CA 92120, USA; erin.r.green@gmail.com (E.G.); lhaase@gmail.com (L.H.); jsazjer@gmail.com (J.S.); 3 Department of Psychiatry, University of California School of Medicine, San Diego, CA 92093, USA

**Keywords:** olfaction, fMRI, obesity, BMI, reward

## Abstract

Obesity has reached epidemic proportions, motivating research into the underlying mechanisms. Olfaction is a powerful mediator of food consumption, and obesity has been associated with altered olfactory sensitivity. The current study used an event-related functional magnetic resonance imaging (fMRI) to examine the central processing of odor in humans to gain insight into the effect of the body mass index (BMI) on the neural processes involved in rating the pleasantness of a food odor during a hunger state and in a satiety state. We hypothesized that, during the hedonic evaluation of food odor, BMI would be associated with differences in brain activation within olfactory and higher order processing areas important for perception, reward, and memory. We report novel findings of a dissociation between the relationship between BMI and activation in reward areas and in olfactory and odor memory areas, i.e., activation in reward areas decreased as BMI increased, whereas activation in primary olfactory and memory regions increased as BMI increased. A greater BMI is associated with decreased activation in the reward and frontal regions, supporting a blunted reward response in obesity. These findings have important potential implications for decision making, response inhibition, and reward-based behaviors that may play key roles as causal and maintenance factors in obesity. In contrast, a greater BMI is associated with an increased activation in the primary olfactory and memory areas, which was observed during a hunger state. These results raise the speculative hypothesis that high BMI may be associated with hyperactivation in the olfactory and memory areas, and that over time, the resulting excitotoxic effects may contribute to neurodegenerative changes in these areas.

## 1. Introduction

Obesity is a global epidemic, with the number of obese adults currently exceeding 1.9 billion worldwide, with recent estimates suggesting that, for example, by 2030, 42% of Americans will be obese [1]. Not only is obesity associated with serious cardiovascular and metabolic risk factors and enormous healthcare costs, it has also been linked to an elevated risk for age-related cognitive impairment and the development of Alzheimer’s disease (AD), which is a significantly increasing public health concern [2,3,4,5]. 

Accordingly, research on causal and maintenance mechanisms for obesity has become increasingly important, especially as decreasing obesity has the potential to aid in the intervention and prevention of AD and normal age-related cognitive declines. One potential focus in this respect has been the physiological and neural systems involved with appetite and eating behaviors, which are thought to play a key role in the development and maintenance of obesity [6,7,8]. 

Olfactory processing is a critical component within these systems. For example, olfaction is known to act as a powerful driver of appetite and nutrient consumption, and olfactory input is also known to be critical in determining the palatability of food in both animals and humans. Olfactory processing can also be influenced by appetite and nutrient consumption. For example, behavioral studies have shown that olfactory processing varies as a function of caloric state (e.g., hunger state, and satiety state, i.e., after a preload). In animal models, rodents display improved odor detection and greater food-odor exploration under fasting conditions, however, odor detection and food-odor exploration decline under the condition of satiety [9,10,11,12]. Research in humans has supported this finding, with studies showing that olfactory perception increases during hunger states and decreases during states of satiety [12,13,14]. 

At the neural level, primary olfactory processing occurs at the level of the olfactory bulb and the piriform cortex, and then proceeds to higher order cognitive processing and reward regions such as the entorhinal cortex, hippocampus, and striatum [15]. These regions integrate executive, memory-based and reward information in order to determine knowledge of, decisions about, and behavioral responses to odors experienced in the environment, particularly in the context of food. 

Importantly, research suggests that many of these regions are negatively affected in obesity. In animals, electrophysiological studies have demonstrated that rodents fed a high fat diet show a marked loss of olfactory sensory neurons, reduced projections from olfactory sensory neurons to the glomeruli in the olfactory bulb, and poorer olfactory discrimination and odor learning [16]. In humans, differences in brain function associated with obesity have been associated with poorer performance on olfactory tasks. For example, research using event-related potentials revealed a positive linear relationship between measures of adiposity (including body mass index (BMI) and waist circumference) and latency of the brain response to odors in older adults performing an odor identification task, suggesting brain response during odor identification was significantly slower with increasing adiposity [17]. Odor identification involves not only the detection of odor, but also the memory for odor and odor names, suggesting the importance of investigating the processing of odor in both sensory and memory areas.

Further research on how differences in olfactory processing at the neural level are related to processing of food-related stimuli within the context of different caloric states (e.g., hunger and satiety) may help elucidate the role of olfactory processing as a potential maintenance mechanism for obesity.

Thus, the main aim of the current study was to gain insight into the relationships between BMI and central olfactory response using event-related functional magnetic resonance imaging (fMRI) to examine brain activation during the hedonic evaluation of a pure odor stimulus in two opposing caloric states: Hunger and satiety. Focusing on activation during a hedonic evaluation task allowed for further investigation of the relationships between BMI and activation in olfactory and higher order memory and reward processing regions, and whether these differ as a function of caloric state. 

We hypothesized that activation would vary as a function of BMI such that greater BMI would be associated with differences in activation within olfactory and higher order processing regions important for reward, executive functioning, and memory. Specifically, based on the literature cited above, we hypothesized that activation in reward areas would decrease with increasing BMI, whereas activation in primary olfactory and memory regions would increase with increasing BMI. Further, it was hypothesized that the relationship between activation and BMI would vary as a function of caloric state, driven by greater activation in the hunger state. 

## 2. Experimental Procedures

### Participants

The participants in the present study were 40 adults, 20 males and 20 females. The means and standard errors for demographic variables are included in Table 1. The participants were assessed for adiposity using waist circumference and BMI. To measure waist circumference, each participant’s waist was measured at the midpoint between the highest point of the iliac crest and the lowest point of the rib cage. Height in inches and weight in pounds were measured during the screening session using a stadiometer and digital scale, respectively. BMI ranged from 19–33 and was calculated by dividing weight in kilograms by height in meters squared after making the metric conversion (see Table 1). Participants were screened for exclusionary criteria, including anosmia and severe hyposmia, upper respiratory infection, nasal stuffiness, chronic cold or sinus conditions, allergies, major head injury and other common sources of smell loss [18], left-handedness, and any contraindications for fMRI (e.g., metal in the body). Participants over the age of 65 were also screened to exclude dementia.

Participants received monetary compensation for participation in the study, which was approved by the Institutional Review Boards at San Diego State University and the University of California, San Diego. All procedures were in accordance with the ethical standards of the institutional research committees and were carried out in accordance with the Declaration of Helsinki.

## 3. Materials: Neuroimaging Stimuli

The stimuli consisted of distilled water and an aqueous (0.02%) solution of citral. While only data from activation to citral were considered for the current publication, the data are part of a larger study in which additional stimuli were also presented. Citral in the mouth evokes a lemon “flavor” that is similar to lemonade, and at this concentration, imparts a lemon odor that is below taste and trigeminal thresholds [19]. Citral was selected as the target stimulus because it is a highly familiar odor, i.e., used in chewing gum, baked goods, candy, non-alcoholic beverages, ice-cream products, etc., and a number of laboratories have employed it successfully in previous neuroimaging [19,20,21] and psychophysical [22,23,24] investigations of retronasal olfactory stimulation. 

## 4. Procedures

### 4.1. Screening Session

Participants completed three separate sessions on different days. The first session served as an opportunity to screen for the exclusionary criteria described above. Screening for anosmia (inability to detect odors) and severe hyposmia (severe impairment in odor detection) was conducted using a standard forced-choice odor threshold detection test on which 0–1 indicates anosmia and 6 is within normal limits (see [25] for the testing procedures). A score of 3 or better was required on the threshold assessment. Participants over the age of 65 were also screened to exclude dementia using the Mattis Dementia Rating Scale [26].

### 4.2. Neuroimaging Sessions

The imaging sessions took place at the University of California, San Diego Center for Functional MRI using a 3T General Electric Signa Excite short bore scanner. The participants were scanned in two separate sessions. In one of the sessions, designated the “hunger” condition, participants were scanned after fasting overnight for a minimum of 12 h. In the second session, designated the “satiety” condition, participants also fasted for a minimum of 12 h prior to the imaging session but consumed a nutritional preload of 26 g of protein and 700 kilocalorie (kcal), by drinking two 350 kcal bottles of Ensure Plus^®^ (Abbott, TX, USA), a nutrition shake produced by Abbott Nutrition (https://ensure.com), 20 min before entering the scanner. The order of hunger and satiety sessions was counterbalanced. 

Participants were asked to report a subjective hunger rating and to provide psychophysical ratings of the pleasantness of the odor stimulus using a modified general Labeled Magnitude Scale (gLMS; [27,28,29]) at several time points outside of the scanner. The modified gLMS measures pleasantness from the strongest unpleasant sensation imaginable to the strongest pleasant sensation imaginable. Participants were asked to compare citral to the “strongest imaginable pleasant sensation of any kind” when rating the stimulus. Hunger was similarly rated on a modified gLMS.

During the hunger scan session, hunger ratings and pleasantness ratings of citral were obtained prior to and after completion of the scan session. During the satiety scan session, participants also reported hunger and pleasantness ratings of citral, which were obtained before and 20 min after preload consumption, as well as after completion of the scan session (see Table 2 for means and standard errors of pre- and post-scan hunger and pleasantness ratings). During fMRI acquisition, pleasantness ratings of the stimuli presented were collected to ensure hedonic evaluation related activation, but in-scan ratings were not a focus of the current study.

### 4.3. Neuroimaging Procedure 

The current study was designed to investigate fMRI activation during the pleasantness evaluation of citral in aqueous solution, to mimic natural flavor perception using a methodology described by Cerf-Ducastel and Murphy [19]. A detailed description of the protocol and system for the oral delivery of the odor stimuli in the fMRI environment that were used in the present study has been outlined by Haase et al. [30]. The critical elements are briefly described below.

**Figure 1:**
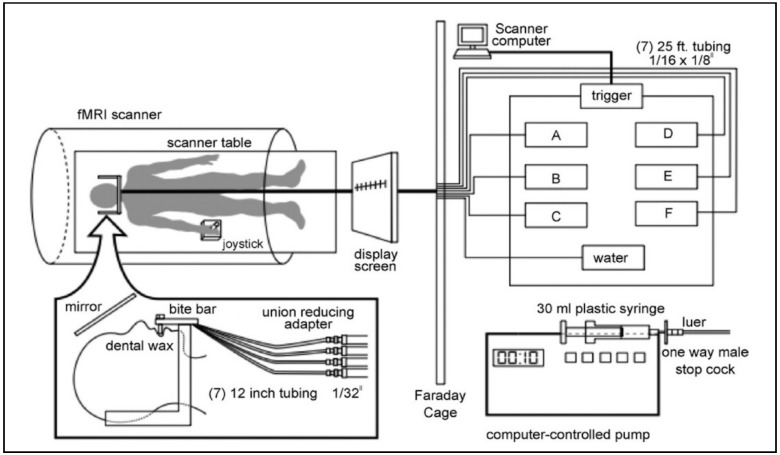
Diagram of computer-controlled stimulus delivery system. Right panel: Each programmable pump held a 30 mL plastic syringe connected to a one-way male stopcock, fitted with a luer that was connected to a 25-foot long plastic tube (Cole Parmer Instrument, PFA, 1/16 inch inside diameter, 1/8 inch outside diameter). The pumps were triggered simultaneously by the scanner computer to deliver the stimuli and water as 0.3 mL of solution in 1 s. The tubes were fastened together with cable ties and were passed through an opening in the Faraday cage in order to reach the subject. Left panel: The 25-foot tubes were connected to 12 inch tubes (Cole Parmer, PFA, 1/32 inch) by a union-reducing adapter. The bite bar was covered with dental wax, which incorporated the tip of individual 12 inch tubes that delivered stimuli to the tip of the tongue. Instructions were displayed on a screen through a computer interface. The instructions were projected onto a mirror placed above the subject’s field of vision. To rate pleasantness, the subject used a joystick to place an arrow on the gLMS. From Haase et al., 2007, reprinted with permission from the *Journal of Neuroscience Methods*.

Each participant underwent two scans: One where intensity was rated and another where pleasantness was rated. The present study included data from only the pleasantness scans. Participants lay supine in the scanner, were directed to remain still and were supplied with padding around the head to minimize head movement. They were fitted with a bite bar containing the tubing for stimulus delivery (Figure 1). The bite bar served to further minimize head movement, including that associated with swallowing, and to allow the tubing for stimulus delivery to rest comfortably between the lips. The target stimulus (citral) and the control (water) were delivered to the tongue of the participant through 25-foot length of tubing, which was connected to programmable syringe pumps located in the fMRI operator room. The pumps were computer-programmed to deliver 0.3 mL in 1 sec from each syringe at the designated time. 

Stimulus presentation was pseudo-random (computer-generated, with the constraint that each stimulus be delivered the same number of times) with a 10 s inter-stimulus interval (ISI). Following each stimulus presentation and rating period, distilled water was first used as a rinse, and a second presentation was used as a neutral baseline for statistical analyses. The odor stimulus was presented 8 times per functional run. The 10 s ISI allowed 1 s for stimulus delivery, 2 s for swallowing (with a cue “please swallow” presented on a screen), and 7 s for providing a magnitude estimate of the pleasantness of the stimulus. A minimum of 30 s elapsed between citral presentations. This procedure was designed to minimize habituation and adaptation.

Two functional scans were performed on each day of scanning. During one of the scans, delivery of the odorant was paired with a pleasantness evaluation task, while intensity evaluation was performed during the other scan. For the current study, only data from pleasantness evaluation scans were analyzed. Participants rated odor pleasantness using a joystick to place a crosshair on a gLMS. A MATLAB program controlled an interactive computer interface that displayed the gLMS on a screen, visible to the participants via a mirror (Figure 1). 

### 4.4. Data Acquisition and Analysis

Structural imaging employed a T1-weighted whole brain MP-RAGE sequence, with a field of view (FOV) of 25 cm and slice thickness of 1 mm. The scan resolution was 1 × 1 × 1 mm^3^, with an echo time (TE) of 4 ms, 136 Locs per slab, and a 15° flip angle. Functional imaging consisted of T2*-weighted images, 24 axial slices, a 19 cm FOV, and a matrix size of 64×64. The spatial resolution used was 3 × 3 × 3 mm^3^, with a TE of 30 ms, a 90° flip angle, and a repetition time (TR) of 2000 ms.

The imaging data were processed using the Analysis of Functional NeuroImages (AFNI) software [31]. Preprocessing consisted of motion correction, temporal and spatial smoothing, and automasking. Deconvolution was then applied to the runs. Deconvolution matches specific blocks of time in the dataset with the impulse response function (IRF) of the hemodynamic response associated with each stimulus presented, enabling precise discrimination of contrasts of interest (e.g., response to citral while rating pleasantness and in a state of hunger). Data from one young female were excluded due to excessive head movement during neuroimaging.

Whole brain group analyses were conducted within AFNI using 3dttest++ with BMI and age as covariates [31]. This process allows the removal of the effect of age (variable of no interest in the current analysis) from the mean of the dataset as well as creating a dataset that includes the effect of BMI (variable of interest) on activation to citral. The output in the dataset reflecting the effect of BMI is the coefficient (slope) of a linear regression and these data were used in the BMI effect results. To control for multiple comparisons, individual voxels that survived a threshold of *p* < 0.015 and were located in a cluster of at least 21 voxels (NN clustering level = 3 corners touching) were considered significantly activated. The minimum cluster size was determined using the AFNI program AlphaSim, which uses Monte Carlo simulation to compute the minimum cluster size necessary to protect the whole-brain probability of false positives at *p* < 0.05, correcting for multiple comparisons [31]. Thus, multiple thresholding steps were conducted in all group analyses. Group statistical maps were corrected for multiple comparisons at the cluster level using Monte Carlo simulations in AFNI’s 3dClustSim program [31]. Monte Carlo simulations calculate the chance of producing a false positive at an overall alpha level of 0.05, based on the voxel connection radius, extent of blurring, individual voxel threshold, and the overall volume of the dataset. 

Region of interest (ROI) analyses were also performed in AFNI using 3dROIstats to test hypotheses regarding the effect of increased adiposity and caloric state on the central processing of olfactory stimuli within specific brain regions. Analyses were restricted to the hunger state to protect against multiple comparisons. The regions were anatomically defined and selected from work based on previous studies and their relevance for olfactory processing (piriform cortex [19,20]) and odor memory processing (entorhinal cortex [32,33,34,35]). Data extracted from the piriform and entorhinal cortices were analyzed using partial correlations between fMRI data and BMI (controlling for age) using the Statistical Package for the Social Sciences (SPSS, version 21).

## 5. Results

### 5.1. Demographic and Psychophysical Measures 

Table 1 provides descriptive statistics for the demographic variables and Table 2 provides the gLMS ratings for hunger and pleasantness.

#### 5.1.1. Hunger Ratings

During the hunger condition, there was a significant effect of time on ratings of hunger, where *F*(1, 35) = 10.22, *p* = 0.003, and partial η^2^ = 0.226, with reported hunger increasing from pre- to post-scan (pre: M = 29.56, SE = 3.68; post: M = 41.41, SE = 4.49). There was also a significant main effect of time/preload on hunger ratings in the satiety condition, *F*(2, 36) = 12.65, *p* < 0.001, partial η^2^ = 0.413. Participants were significantly less hungry post-preload (M = 9.23, SE = 2.44) and post-scan (M = 12.10, SE = 2.58) than after the 12-h fast (M = 29.31, SE = 3.66), indicating the nutritional preload was successful in decreasing hunger prior to entering the scanner. 

#### 5.1.2. Pleasantness Ratings

There were no main effects of time on pleasantness ratings in either the hunger condition, *F* (1, 38) = 0.80, *p* = 0.378, partial η^2^ = 0.021; or in the satiety condition, *F* (2, 36) = 1.79, *p* = 0.182, partial η^2^ = 0.090.

### 5.2. Voxelwise Imaging Analyses: Activation during the Pleasantness Rating of Citral vs. Water

Table 3, Table 4, Table 5, Table 6 and Table 7 comprehensively report activation in all of the contrasts. In the text below we highlight activations in those areas that are of particular relevance for the hypotheses.

#### 5.2.1. Hunger Condition 

**Figure 2:**
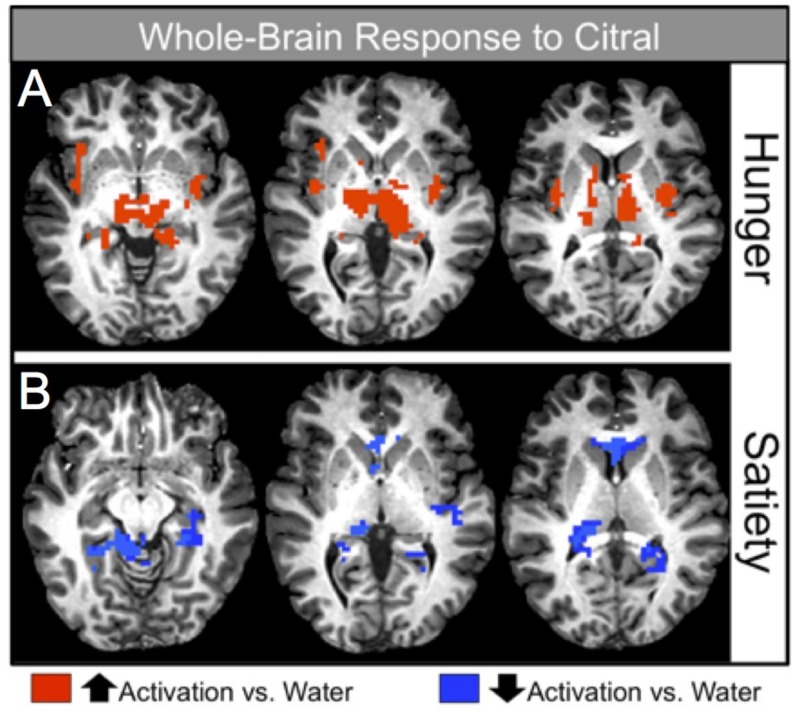
Image depicting significant whole-brain activation in response to the odorant, citral, during the hunger and satiety conditions. Red coloring indicates positive activation, blue coloring indicates negative activation (relative to activation in response to water).

Table 3 and Figure 2A display significant activation seen during the pleasantness rating of citral in the hunger condition after controlling for age and BMI (*p* < 0.001). Increased activation relative to baseline (water) was demonstrated in the odor and olfactory processing regions (piriform and entorhinal cortices, and thalamus) and in areas important in odor/memory (parahippocampal gyrus, hippocampus), reward processing (amygdala, caudate nucleus, putamen) and energy regulation (hypothalamus). The cerebellum and additional regions were also activated (see Table 3). 

#### 5.2.2. Satiety Condition

Table 4 and Figure 2B display the significant activation seen during the pleasantness rating of citral in the satiety condition after controlling for age and BMI. Of particular interest, significant decreases in activation were found in reward regions (the caudate), sensory and olfactory processing areas (thalamus, entorhinal cortex), and in memory processing regions (hippocampus, parahippocampal gyrus). Refer to Table 4 for a complete list of brain regions that exhibited a reduction in activation relative to baseline (*p* < 0.001). 

#### 5.2.3. Hunger-Satiety Contrast

Cortical response in the hunger condition was compared to activation in the satiety condition to determine the effect of caloric state on brain response during the pleasantness evaluation of citral after controlling for age and BMI. Overall, activation was significantly greater in the hunger condition relative to the satiety condition. Robust significant differences in activation were demonstrated in the sensory (insula, thalamus), frontal (inferior frontal gyrus), reward (caudate, lentiform nucleus, orbitofrontal cortex (OFC) BA 47, putamen, amygdala), and odor/memory/temporal areas (entorhinal cortex, hippocampus, parahippocampal gyrus, superior temporal gyrus). A complete list of activated brain regions is shown in Table 5 (*p* < 0.001).

### 5.3. Voxelwise Imaging Analyses: Activation as a Function of Body Mass Index

#### 5.3.1. Hunger Condition

**Figure 3:**
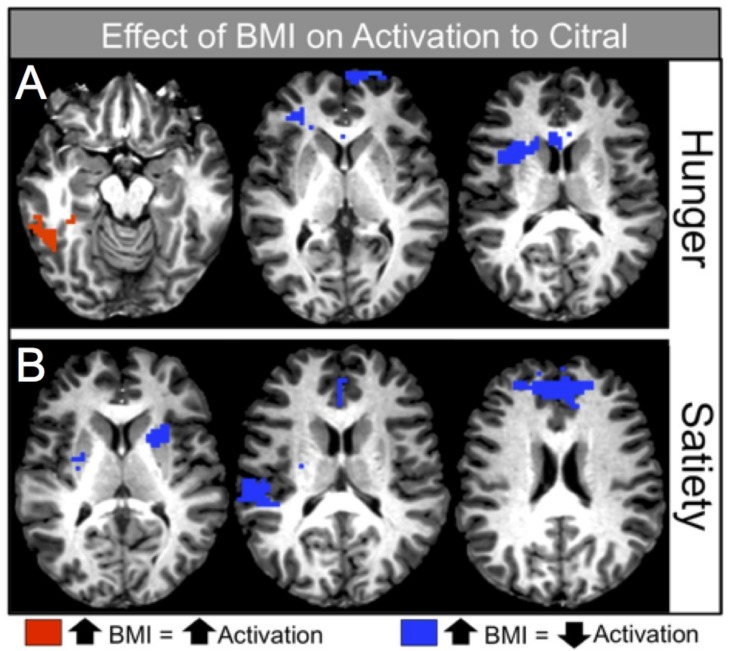
Image depicting strength of the association between BMI and brain activation in response to the odorant, citral, during the hunger and satiety conditions. Red coloring indicates significant positive associations between BMI and activation (as BMI increased, activation increased), blue coloring indicates significant negative associations between BMI and activation (as BMI increased, activation decreased).

Table 6 and Figure 3A display the significant relationships between BMI and voxelwise activation in the hunger condition. There were both positive and negative relationships between BMI and brain activation during pleasantness evaluation of citral (controlling for age). Of particular interest, as BMI increased, activation decreased in sensory areas such as the anterior insula, frontal regions, including the dorsolateral prefrontal cortex (DLPFC) and BA 10, and in reward processing centers of the brain (caudate nucleus). In contrast, cortical response increased in other sensory areas, such as the posterior insula, as well as in memory and semantic processing-related regions such as the fusiform and parahippocampal gyri. For a complete list of regions that were positively and negatively associated with BMI during the hunger condition, see Table 6 (*p* < 0.015).

#### 5.3.2. Satiety Condition

Table 7 and Figure 3B display the significant relationships between BMI and voxelwise activation in the satiety condition. In this condition, as BMI increased, activation decreased in the sensory (insular cortex), reward (caudate nucleus, lentiform nucleus, putamen), and frontal areas (BA 10), as well as in cingulate gyrus. For a complete list of regions that were negatively associated with BMI during the satiety condition, see Table 7 (*p* < 0.015).

### 5.4. ROI Analyses

**Figure 4:**
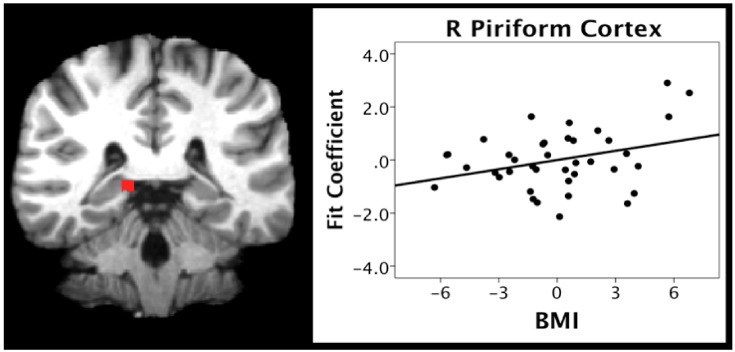
Scatterplot depicting the correlation between BMI and activation in piriform cortex in the hunger condition.

**Figure 5:**
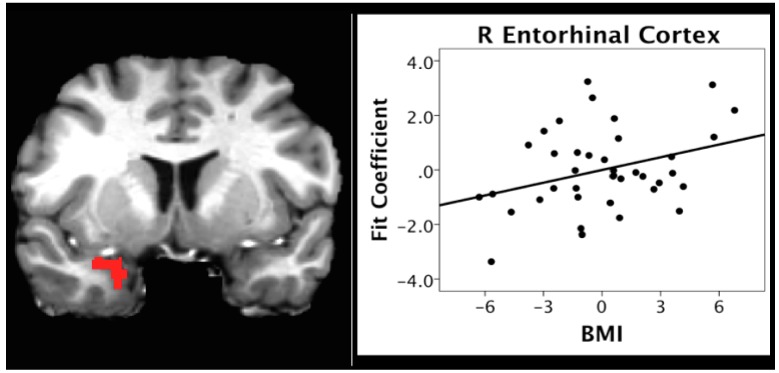
Scatterplot depicting the correlation between BMI and activation in entorhinal cortex in the hunger condition.

We also investigated the correlation between BMI and activation during the pleasantness evaluation of citral in the hunger condition. We focused on two anatomically-defined regions due to their importance in olfactory (the piriform cortex) and odor memory (entorhinal cortex) processing. BMI scores were positively correlated with activation in the right piriform (*r* = 0.329, *p* = 0.044) and entorhinal (*r* = 0.334, *p* = 0.040) cortices (Figure 4 and Figure 5, respectively). 

## 6. Discussion

The present study investigated the relationship between BMI and brain activation during the pleasantness rating of a pure odor stimulus within two caloric states: Hunger and satiety. We hypothesized that fMRI activation in the olfactory and higher-order processing regions would vary as a function of caloric condition and BMI. 

We found a dissociation between the relationship between BMI and activation in reward areas and in olfactory and odor memory areas, such that activation in reward areas decreased as BMI increased, whereas activation in primary olfactory and memory regions increased as BMI increased. Lower activation in the reward and frontal regions, in association with higher BMI, supports previous findings showing a blunted reward response in obesity that has been linked to changes in decision making, response inhibition, and reward-based behaviors, which may serve as causal and maintenance factors in obesity. In contrast, increasing BMI associated with increased activation in the primary olfactory and memory areas was observed during hunger.

In order to investigate fMRI activation in response to odor under conditions that mimic natural flavor perception and ingestion, the present study used a methodology described by Cerf-Ducastel and Murphy [19], in which the odorant is delivered orally via an aqueous solution. Importantly, significant activation was seen in the piriform (primary olfactory cortex), an area not reported to be active in previous studies from our group using gustatory stimuli in this paradigm [36,37,38,39,40,41]. These results support the utility of the delivery of odor stimuli via aqueous solutions into the oral cavity, suggesting that brain activation in response to an odor stimulus acting through the retronasal pathway is similar to the brain activation previously shown to occur in response to gustatory stimuli. 

## 7. Activation to Odor as a Function of Caloric Condition

Independent of BMI and age, the processing of olfactory information in the human brain was highly dynamic and dependent on caloric state (hunger vs. satiety). Overall brain activation was greater in the hunger state than in the satiety state. This was true in the brain regions important for primary and higher order olfactory/sensory processing, reward processing, and memory (see Table 5). Many of these regions are associated with appetite, palatability and the hedonic valuation of nutritive stimuli and food consumption [42,43]. 

These results align with previous findings indicating that brain activation to taste stimuli is greater in hunger states than sated states [38]. It is also consistent with studies that investigated the effects of the judging, liking and wanting of food-related odor [44]. Finally, these findings also support the translational potential of research in animals that suggest that brain activity is greater during hunger than satiety, and the modulating influence that obesity and diet may have over these effects [12,15,45,46].

## 8. BMI and Reward Activation 

Within both caloric conditions, decreased activation was seen with increasing BMI in brain regions associated with reward processing. For example, increased BMI was associated with decreased activation of the right caudate nucleus in the hunger condition (Table 6, Figure 3A, right), and decreased activation of the bilateral caudate and putamen in the satiety condition (Table 7, Figure 3B, left). 

Negative associations between obesity and reward response to nutritive stimuli have also been reported in overweight adolescent females. Studies have shown increased activation in the gustatory and reward circuitry in response to the anticipation of a chocolate milkshake and decreased gustatory and reward circuitry activation in response to the intake of a chocolate milkshake [46,47]. The present findings suggest that decreased reward activation occurs not only in response to complex food stimuli, but also to a simple food odor stimulus. 

The relationship between BMI and the caudate nucleus was found in both hunger and satiety conditions. Recent research in humans has linked increased BMI with a decreased caudate response to sucrose, a pure taste stimulus [43], and to a milkshake, which stimulates both olfaction and taste [46,48]. The present results support a decreased caudate response associated with a pure olfactory stimulus. Importantly, the caudate and its connections to other brain regions within the basal ganglia, limbic structures, and the prefrontal cortex are highly associated with reward-related dopaminergic activity. While dopamine levels were not measured directly in the present study, the BOLD response measured during fMRI is directly coupled to dopamine activity [49,50]. 

Thus, these findings lend support to theories suggesting that increasing BMI may be associated with changes in dopaminergic function. In particular, obesity has been associated with decreased striatal D_2_ receptor availability, and this abnormality in dopamine function has been linked to reward deficiency syndrome [51,52,53]. Reward deficiency syndrome refers to the predisposition to seek to engage in additional rewarding behaviors to compensate for inefficient dopamine functioning [52]. In the case of overweight and obese individuals, the rewarding behavior of choice may be over-eating, or eating foods high in sugar or fat content. 

## 9. BMI and Frontal Activation

The present results also indicated a negative relationship between BMI and regions critical for decision-making about rewarding stimuli, such as the frontopolar prefrontal cortex (BA 10; Table 6 and Table 7). BA 10 is hypothesized to play a role in binary choices about, and the valuation of, olfactory and reward stimuli, as well as with the ease of and confidence in decision making during olfactory-based choices [54,55]. Activation in BA 10 has also been shown to correlate with goal-oriented decisions about food cues and food stimuli [56,57]. 

We might speculate that decreased response in BA 10 would be associated with poor food choice in obesity, especially when coupled with decreases in reward that increase risk-taking and reinforcement-seeking behavior. Notably, dysfunction in the ventro-lateral regions of the prefrontal cortex, such as the inferior frontal gyrus, has also been associated with impulsivity and behavioral factors such as increased risk taking in decision making [58,59]. Studies of obesity have linked impulsivity and risky decision making to weight gain and dysfunctional eating behaviors, such as eating in the absence of hunger, binge eating, and emotionally-motivated eating [47,60]. Thus, the present findings suggesting decreased activation in both reward and prefrontal brain regions with increasing BMI highlight a potential neuro-behavioral mechanism for obesity. 

## 10. Increased Activation in Olfactory Sensory and Memory Regions during Hunger

In contrast to the negative relationship between BMI and activation within the reward and frontal regions across caloric conditions, the present study found a positive relationship between BMI and activation within regions important for olfactory and higher-order processing, such as odor memory, during the hunger condition (Table 6). ROI analyses supported the whole brain analyses, indicating a positive relationship between BMI and activation in regions critical to memory and olfactory processing, specifically the entorhinal and piriform cortices (see Figure 4 and Figure 5). Similar findings have been reported by studies in animals showing the hyper-responsivity of sensory processing brain systems in obesity, which has been associated with behaviors such as over-eating [12]. 

Hyperactivity associated with increasing obesity may have important implications, as it is theorized that long-term neuronal hyperactivity within a brain region may result in excitotoxic effects that have been implicated as a potential mechanism for the cognitive declines reported in obesity and obesity-related risk factors such as insulin resistance and high-fat diets [4,5,61,62]. Obesity-related excitotoxicity may also serve as a mechanism for age-related cognitive declines [63] and neurodegenerative conditions such as AD [64,65,66,67,68].

For example, animal models have found that hyperactivity in the hippocampus and the lateral entorhinal cortex has been associated with the production and secretion of amyloid β (Aβ) and Aβ precursor protein (AβPP) metabolites [66,69], important in the development of AD. In humans, hyperactivation in the hippocampus, which maintains direct connections to the entorhinal cortex, has been previously reported in cases of very early and moderate mild cognitive impairment (MCI) [70,71]. Although the role and the trajectory of hyperactivity in AD is uncertain [66,72], it has been reported to be associated with atrophy and clinical progression in amyloid positive patients with MCI, a percentage of whom will go on to develop AD [73]. 

The present findings of an association between increasing BMI or waist circumference and hyperactivity in regions critical for olfactory processing are particularly noteworthy, given that neuropathology in the olfactory bulbs and entorhinal cortex manifests very early in AD [74,75,76,77] and olfactory function has been shown to decline in the preclinical stages of the disease [67,68,78,79]. 

**Figure 6:**
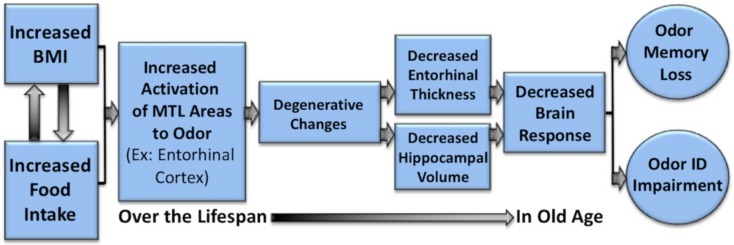
Potential mechanism whereby increased BMI is associated with the hyperactivation of the olfactory and mesial temporal areas (e.g., entorhinal cortex and hippocampus), thus contributing to the deterioration of areas critical to olfactory and memory processing in aging and neurodegenerative disorders, particularly Alzheimer’s disease (AD). Degeneration in these areas will be reflected in decreased brain response and impaired performance on olfactory tasks, such as odor identification and odor memory.

Thus, these data would support a speculative hypothesis that the hyperactivation of olfactory and odor memory processing regions, including the piriform and entorhinal cortices, may be one underlying mechanism for the effects of high BMI on olfactory processing, and on the degeneration of the entorhinal cortex over time in individuals with a high BMI [67]. If this is the case, it would represent one potential mechanism whereby the BMI may contribute to deterioration of areas critical to olfaction and memory function with aging and neurodegenerative disorders, particularly AD (see Figure 4, Figure 5 and Figure 6). Future research is warranted to systematically examine this hypothesis in longitudinal cohorts. 

In summary, we present novel findings of a dissociation between the relationship between BMI and functional brain activation in reward regions, and activation in primary olfactory and memory regions during hedonic evaluation of an odor stimulus: Activation in reward areas decreased as BMI increased in both the hunger and satiety conditions, whereas activation in primary olfactory and higher order processing and memory regions increased as BMI increased during the hunger condition. These findings expand upon previous reports of the effects of overweight and obesity-related variables like adiposity and BMI on brain activation in reward areas in response to gustatory stimuli [43] and visual food-related stimuli [42], suggesting that these patterns of activation may be associated with food-related stimuli in general, and are not specific to a sensory modality. A blunted reward and frontal response during the hedonic evaluation of a food-related odor has important potential implications for decision making, response inhibition, and reward-based behaviors such as binge-eating and eating-when-full, which may play key roles as causal and maintenance factors for obesity. Further research investigating how the functional response to olfactory stimuli differs in overweight and normal weight individuals, as well as the mechanisms underlying these changes, is warranted. 

## Figures and Tables

**Table 1:** Demographics.

Demographic Variable	Mean	SE
Age (years)	48.9	4.0
Weight (lbs)	162.9	4.3
Body Mass Index (BMI) (kg/m^2^)	26.3	0.6
Waist Circumference (cm)	90.3	1.8

**Table 2:** Descriptive statistics: Hunger ratings, odor pleasantness ratings and odor threshold.

Psychophysical Variable	Mean	SE
Hunger After Fast ^a^	35.7	3.4
Hunger Post-Preload ^a^	2.7	0.7
Pleasantness Pre-Scan (Hunger Scan) ^a^	52.0	1.3
Pleasantness During Scan (Hunger Scan) ^a^	46.8	1.3
Pleasantness Post-Scan (Hunger Scan) ^a^	50.9	1.7
Pleasantness Pre-Scan (Satiety Scan) ^a^	49.0	1.9
Pleasantness Post-Preload (Satiety Scan) ^a^	48.6	1.0
Pleasantness During Scan (Satiety Scan) ^a^	47.3	1.8
Pleasantness Post-Scan (Satiety Scan) ^a^	49.1	1.0
Odor Threshold Right Nostril ^b^	6.5	0.2
Odor Threshold Left Nostril ^b^	6.6	0.3

^a^ Rating on a modified general labeled magnitude scale (gLMS) (0–100). ^b^ Score on odor butanol detection task (0–9).

**Table 3:** Whole-brain activation to odor during the hunger condition.

*p* < 0.001, Min. Cluster = 11 Voxels			TLRC		*t*	Voxels
Region	Hem.	x	y	z	Stat.	In Clust.
Thalamus	L	−6	−20	4	8.03	1115
Thalamus	R	10	−17	4	6.69	
Cerebellum	L	−10	−31	−7	6.58	
Parahippocampal Gyrus	L	−16	−35	0	6.3	
Piriform Cortex/BA 27	L	−20	−30	−5	6.27	
Substantia Nigra	L	−7	−17	-7	6.07	
Lentiform Nucleus	R	18	−9	5	5.82	
Fusiform Gyrus	L	−30	−41	−14	5.63	
Cerebellum	R	9	−31	−7	5.57	
Medial Globus Pallidus	R	15	−7	1	5.48	
Posterior Cingulate	L	−15	−13	11	5.43	
Substantia Nigra	R	8	−17	−7	5.28	
Red Nucleus	R	6	−22	−4	5.25	
Red Nucleus	L	−6	−22	−4	5.16	
Hypothalamus	R	8	−4	−5	5.09	
Caudate Body	R	11	11	10	4.99	
Lingual Gyrus	L	−19	−40	−2	4.97	
Lentiform Nucleus	L	−14	−4	5	4.94	
Caudate	L	−7	5	11	4.89	
Caudate Body	L	−6	5	10	4.89	
Caudate Head	R	11	12	4	4.84	
Caudate Tail	R	20	−31	15	4.74	
Caudate	R	11	8	11	4.7	
Anterior Insula	R	37	19	2	5.97	161
Posterior Insula	R	40	−2	−8	5.64	
Amygdala	R	24	−2	−11	5.45	
BA 13	R	41	1	−4	5.4	
Superior Temporal Gyrus	R	40	−3	−9	5.34	
Parahippocampal Gyrus	R	26	2	−13	4.78	
Inferior Frontal Gyrus	R	37	23	0	4.72	
OFC BA 47	R	37	23	0	4.72	
Hippocampus	R	26	−11	−15	4.61	
Claustrum	R	30	17	1	3.53	
Subcallosal Gyrus	R	25	8	−11	2.68	
Putamen	L	−27	−7	13	5.6	92
Anterior Insula	L	−38	2	0	5.04	
Amygdala	L	−22	−8	−9	4.89	
Posterior Insula	L	−36	−19	13	4.8	
BA 13	L	−40	−13	13	4.77	
Lentiform Nucleus	L	−22	−7	−8	4.72	
Parahippocampal Gyrus	L	−25	−13	−10	4.69	
Postcentral Gyrus	L	−49	−13	15	4.09	
Rolandic Operculum/BA 43	L	−49	−13	15	4.09	
Hippocampus	L	−28	−13	−11	3.13	
Claustrum	L	−35	−4	−8	2.96	
Hippocampus	R	29	−33	−6	6.12	90
Piriform Cortex/BA 27	R	22	−31	−6	5.83	
Parahippocampal Gyrus	R	20	−31	−4	5.71	
Thalamus	R	20	−32	0	5.1	
Entorhinal Cortex/BA 28	R	21	−28	−6	5.1	
Fusiform Gyrus	R	31	−43	−12	4.99	
Culmen	R	28	−47	−12	4.61	

TLRC x y z: Talairach coordinates, Hem: Hemisphere, L: Left hemisphere, R: Right hemisphere, BA: Brodmann’s Area, OFC: Orbitofrontal cortex.

**Table 4:** Whole-brain activation to odor during the satiety condition.

*p* < 0.001, Min. Cluster = 11 Voxels			TLRC		*t*	Voxels
Region	Hem.	x	y	z	Stat.	In Clust.
Parahippocampal Gyrus	L	−26	−38	−9	−4.75	799
Caudate Tail	R	27	−35	9	−4.63	
Caudate Body	L	−13	−13	22	−4.52	
Thalamus	L	−10	−26	16	−4.47	
Thalamus	R	13	−29	5	−4.39	
Cerebellum	R	5	−46	−31	−4.38	
Posterior Insula	L	−40	−16	6	−4.35	
BA 13	L	−41	−16	5	−4.35	
Cerebellum	L	−4	−50	−31	−4.3	
Parahippocampal Gyrus	R	27	−29	−13	−4.22	
Fusiform Gyrus	R	28	−53	−8	−3.96	
Claustrum	L	−32	−9	−2	−3.95	
Lentiform Nucleus	L	−31	−10	0	−3.95	
Putamen	L	−30	−10	−2	−3.95	
Amygdala	L	−22	−9	−15	−3.93	
Entorhinal Cortex/BA 28	R	19	−16	−12	−3.92	
Caudate Body	R	14	−14	19	−3.89	
Superior Temporal Gyrus	L	−44	−20	4	−3.87	
Caudate	L	−35	−23	−7	−3.85	
Substantia Nigra	R	13	−22	−9	−3.83	
Fusiform Gyrus	L	−27	−41	−11	−3.47	
Caudate Tail	L	−33	−32	−4	−3.3	
Entorhinal Cortex/BA 28	L	−18	−15	−11	−3.14	
Hippocampus	L	−29	−16	−11	−2.5	
Hippocampus	R	31	−34	−5	4.6	
Anterior Cingulate	R	2	23	11	−5.24	102
Caudate	R	15	22	7	−4.57	
Caudate Body	R	5	6	9	−4.52	
Caudate Head	R	8	14	1	−4.23	
Caudate	L	−7	17	14	−4.06	
Caudate Body	L	−8	16	14	−4.06	
Caudate	L	−19	−40	11	−5.49	52
Parahippocampal Gyrus	L	−27	−50	11	−4.88	

TLRC x y z: Talairach coordinates, Hem: Hemisphere, L: Left hemisphere, R: Right hemisphere, BA: Brodmann’s Area.

**Table 5:** Differences in activation to odor between caloric conditions (hunger, satiety).

*p* < 0.001, Min. Cluster = 11 Voxels			TLRC		*t*	Voxels
Region	Hem.	x	y	z	Stat.	In Clust.
Hippocampus	R	30	−26	−9	6.18	2107
Parahippocampal Gyrus	R	26	−26	−12	6.03	
Putamen	L	−30	−10	3	6	
Claustrum	L	−33	−4	5	5.71	
Inferior Frontal Gyrus	R	31	19	−7	5.68	
Posterior Cingulate	L	−10	−48	14	5.56	
Caudate	R	27	−35	9	5.44	
Cerebellum	R	10	−32	−22	5.39	
Entorhinal Cortex/BA28	L	22	−27	−7	5.36	
Caudate Tail	R	28	−35	10	5.34	
Amygdala	R	24	−7	−15	5.33	
Entorhinal Cortex/BA28	R	22	−12	−16	5.3	
Claustrum	R	35	−4	7	5.27	
Parahippocampal Gyrus	L	−25	−25	−11	5.24	
Anterior Insula	L	−33	−4	9	5.23	
Hypothalamus	R	7	−4	−5	5.23	
Thalamus	L	−12	−19	8	5.21	
Posterior Cingulate	R	11	−52	14	5.2	
Fusiform Gyrus	L	−31	−41	−12	5.19	
Fusiform Gyrus	R	32	−47	−14	5.17	
Lentiform Nucleus	R	30	−16	2	5.16	
Superior Temporal Gyrus	R	37	2	−10	5.16	
Putamen	R	29	−16	3	5.16	
Lentiform Nucleus	L	−31	−5	2	5.14	
Anterior Insula	R	37	−7	9	5.11	
Thalamus	R	11	−19	8	5.1	
Amygdala	L	−23	−9	−15	5.05	
Caudate	L	−7	7	9	5.02	
Caudate Body	L	−7	7	9	5.02	
BA 13	L	−41	2	−2	5	
Hippocampus	L	−29	−18	−11	4.94	
Cerebellum	L	−10	−31	−22	2.72	
Inferior Frontal Gyrus	R	23	16	−10	5.41	12
Lentiform Nucleus	R	22	13	−10	5.32	
Claustrum	R	29	11	−8	5.1	
Anterior Insula	R	28	19	−8	5.05	
OFC BA 47	R	30	20	−8	5.05	
BA 13	R	29	19	−8	5.02	
Subcallosal Gyrus	R	26	5	−10	2.16	
Lentiform Nucleus	L	−22	−7	−8	4.72	
Parahippocampal Gyrus	L	−25	−13	−10	4.69	
Postcentral Gyrus	L	−49	−13	15	4.09	
Rolandic Operculum/BA 43	L	−49	−13	15	4.09	
Hippocampus	L	−28	−13	−11	3.13	
Claustrum	L	−35	−4	−8	2.96	
Hippocampus	R	29	−33	−6	6.12	90
Piriform Cortex/BA 27	R	22	−31	−6	5.83	
Parahippocampal Gyrus	R	20	−31	−4	5.71	
Thalamus	R	20	−32	0	5.1	
Entorhinal Cortex/BA 28	R	21	−28	−6	5.1	
Fusiform Gyrus	R	31	−43	−12	4.99	
Culmen	R	28	−47	−12	4.61	

TLRC x y z: Talairach coordinates, Hem: Hemisphere, L: Left hemisphere, R: Right hemisphere, BA: Brodmann’s Area.

**Table 6:** The effect of BMI on whole-brain activation to odor in the hunger condition.

*p* < 0.15, Min. Cluster = 21 Voxels		TLRC			Regr.	Voxels
Region	Hem.	x	y	z	Coef.	In Clust.
Anterior Cingulate	R	5	23	17	−0.45	116
Inferior Frontal Gyrus	R	32	31	15	−0.34	
Medial Frontal Gyrus	R	28	31	15	−0.32	
Anterior Cingulate	L	−4	23	17	−0.29	
BA 46 DLPFC	R	37	37	11	−0.22	
BA 10	R	14	40	11	−0.20	
BA 13	R	36	20	13	−0.17	
Anterior Insula	R	37	22	12	−0.16	
Superior Frontal Gyrus	R	27	37	12	−0.16	
Middle Frontal Gyrus	R	23	44	−1	−0.12	
Superior Frontal Gyrus	L	−19	68	11	−1.24	55
Medial Frontal Gyrus	L	−13	65	5	−0.41	
Middle Frontal Gyrus	L	−23	62	8	−0.41	
BA 10	L	−13	65	5	−0.41	
Inferior Temporal Gyrus	R	53	−49	−13	0.30	54
Fusiform Gyrus	R	36	−34	−16	0.20	
Middle Temporal Gyrus	R	55	−53	−7	0.20	
Parahippocampal Gyrus	R	34	−34	−12	0.11	
Posterior Insula	R	41	−19	−1	0.13	38
Claustrum	R	36	−22	3	0.13	
BA 13	R	39	−22	3	0.13	
Superior Temporal Gyrus	R	45	−38	5	0.12	
Middle Temporal Gyrus	R	47	−25	−7	0.11	
Anterior Insula	R	32	17	17	−0.38	32
Caudate	R	17	17	17	−0.24	
Caudate Body	R	16	14	17	−0.18	
Claustrum	R	29	9	17	−0.16	
BA 13	R	34	5	17	−0.15	
Cerebellum	R	34	−64	−34	1.01	23

TLRC x y z: Talairach coordinates, Hem: Hemisphere, L: Left hemisphere, R: Right hemisphere, BA: Brodmann’s Area, DLPFC: Dorsolateral prefrontal cortex.

**Table 7:** The effect of BMI on whole-brain activation to odor in the satiety condition.

*p* < 0.15, Min. Cluster = 21 Voxels		TLRC			Regr.	Voxels
Region	Hem.	x	y	z	Coef.	In Clust.
Anterior Cingulate	L	−25	59	20	−0.51	133
Medial Frontal Gyrus	L	−3	56	19	−0.48	
Medial Frontal Gyrus	R	2	56	19	−0.48	
Middle Frontal Gyrus	L	−20	57	21	−0.46	
Superior Frontal Gyrus	L	−14	56	21	−0.42	
Superior Frontal Gyrus	R	13	51	21	−0.42	
BA 10	R	5	57	21	−0.41	
Middle Frontal Gyrus	R	24	54	20	−0.36	
BA 10	L	−4	58	21	−0.35	
Anterior Cingulate	R	5	38	23	−0.26	
Precentral Gyrus	R	50	−7	26	−0.40	47
Postcentral Gyrus	R	49	−4	26	−0.38	
Postcentral Gyrus	R	62	−19	17	−0.31	46
Superior Temporal Gyrus	R	58	−29	17	−0.24	
Transverse Temporal Gyrus	R	53	−22	13	−0.18	
BA 13	R	47	−23	18	−0.18	
Anterior Insula	R	50	−19	17	−0.17	
Caudate	R	23	−31	20	−0.17	38
Caudate Tail	R	18	−25	19	−0.15	
Anterior Insula	R	29	−22	19	−0.12	
Lentiform Nucleus	R	30	−4	8	−0.11	
Putamen	R	27	−5	9	−0.10	
Claustrum	R	32	−1	8	−0.08	
Inferior Parietal Lobule	L	−37	−25	29	−0.31	28
Postcentral Gyrus	L	−37	−25	30	−0.31	
Caudate	L	−25	−22	29	−0.23	
Cingulate Gyrus	L	−10	−19	29	−0.23	
Caudate	L	−19	11	11	−0.17	21
Claustrum	L	−28	17	11	−0.14	
Anterior Insula	L	−30	17	12	−0.14	
Caudate Body	L	−16	11	12	−0.14	
Lentiform Nucleus	L	−19	10	5	−0.13	
Putamen	L	−19	11	5	−0.13	

TLRC x y z: Talairach coordinates, Hem: Hemisphere, L: Left hemisphere, R: Right hemisphere, BA: Brodmann’s Area, DLPFC: Dorsolateral prefrontal cortex.
